# Reliability of blood tests taken from the peripheral intravenous catheter

**DOI:** 10.1097/MD.0000000000029268

**Published:** 2022-07-15

**Authors:** Idan Ben Shabat, Michal Hoffman Ben Shabat, Shai Ben Abraham, Iftach Sagy, Gal Tsaban, Merav Cohen-Lahav, Gil Goldinger, Michal Peled, Carmi Bartal

**Affiliations:** a Internal Medicine Division, Soroka University Medical Center, Ben-Gurion University of the Negev, Beer-Sheva, Israel; b Division of Biochemistry Laboratory, Soroka University Medical Center, Ben-Gurion University of the Negev, Beer-Sheva, Israel.

**Keywords:** direct venipuncture stab, laboratory blood examinations, peripheral venous catheter, sampling methods

## Abstract

We aimed to compare the reliability of laboratory blood tests using 2 sampling methods, via a peripheral venous catheter (PVC) vs direct venipuncture stab (DVS), we evaluated the effect of time elapsed since PVC insertion, PVC diameter, and administration of saline and/or antibiotic infusion through PVC on the blood test results.

A prospective comparative study was conducted between May 2018 and July 2019. Patients aged ≥ 18 years and admitted to our department with a 20G/22G PVC inserted within the last 24 hours were enrolled.

Blood samples were collected from each participant in the morning, and a second sample was drawn using PVC. Dependent variables included the percentage of hemolysis, failure rate, complete blood count, biochemical testing parameters, and coagulation functions.

A total of 211 patients participated in the study. In total, 237 blood tests were conducted, of which 167 were performed on day 1 and the remaining on day 2, with a second blood sample collected from 26 patients on day 2. Twenty-one participants received 22G PVC, and 23 participants received active infusion. No significant differences were found in failure rates when each subgroup was compared with the primary day 1 group. The intraclass correlation coefficient indicated significant correlations among all the indices in all groups.

Both blood sampling methods (PVC and direct venipuncture) can be used interchangeably for routine laboratory tests on days 1 and 2 after PVC insertion using 20G/22G PVC or infused PVC.

## 1. Introduction

Many hospitalized patients require invasive diagnostic tests, including blood tests. Hospitalized patients usually require the insertion of a peripheral venous catheter (PVC) in the emergency room. At insertion and before infusion, 2 blood samples were taken directly from the catheter for routine testing. Subsequently, any additional blood test was performed via a direct venipuncture stab (DVS) or “butterfly stab, ” despite the presence of permanent venous access through PVC.

Prioritization of DVS over drawing blood from an existing venous catheter is due to concerns that catheter use causes hemolysis in the sample, thus rendering the results unreliable. Indeed, several studies have shown that PVC blood sampling leads to higher hemolysis rates than DVS sampling.^[1–3]^ However, other factors may decrease the hemolysis rate. A few trials have shown that larger venous catheter diameter and more proximal catheter position (at the antecubital fossa vs the back of the hand) may reduce hemolysis rate.^[4,1]^ In addition, use of syringe for manual extraction of the sample from the catheter also results in lower hemolysis rate compared with vacuum pumping (with a vacutainer).^[2,5,6]^ Additional decrease in hemolysis rate can be achieved by drawing a “dead space” sample from the PVC.^[6]^ Even if no hemolysis occurs, there is concern that because time has elapsed since catheter insertion and its use (drug administration and infusion through PVC), the results will not be reliable^[8–10,16,17]^ Blood sampling via PVC for routine tests (complete blood count [CBC], blood chemistry, coagulation, creatinine level, liver function, and troponin level) is a reliable method.^[7–13]^ However, these studies had several limitations: a low number of participants and indicators examined and no reference to failure in drawing blood through PVC.

The primary goal of our study was to compare the reliability of routine laboratory blood tests achieved using 2 different methods of blood sampling: DVS and PVC. In addition, we examined 3 other potential causes affecting the reliability of blood test results: time elapsed since catheter insertion (1 and 2 days after insertion), PVC diameter, and effect of saline administration and/or antibiotic infusion through PVC.

### 1.1. Design and setting methods

This was a prospective comparative study conducted at the Internal Medicine Department E, Soroka Medical Center, Be*’*er Sheva, Israel, between May 2018 and July 2019. Soroka Medical Center is a tertiary hospital with ~120 (211) 0 beds and ~100,000 hospitalizations per year. The institution provides medical services to 1 million residents of the region.

### 1.2. Study population and sample size

The study population comprised patients aged ≥ 18 years admitted to the Internal Medicine Department E and who had undergone 20G/22G PVC insertion within the last 24 hours. Patients with language difficulties, cognitive disorders, or an inability to provide informed consent, and those with arteriovenous shunts were excluded. Patients diagnosed with HIV or hepatitis A, B, or C were also excluded.

Sample size calculations were based on serum hemoglobin levels, with a mean of 11.8 ± 0.9 and 11.8 ± 1.0 mg/dL for DVS and PVC, respectively.^[15]^ A difference of up to 0.06 was considered negligible, and correlation coefficient between measurements was assumed to be 0.99. Considering a 2-sided significance level of α = 5% and a potential noninclusion of 15% of the samples, a total of 200 paired blood test samples were required to detect this effect with a power of 90%.

### 1.3. Data collection instruments and procedure

We included more than the planned number of participants (211), as all patients who agreed to participate were included. Blood tests were performed after obtaining informed consent from the participants. All blood samples were drawn by 6 blood collectors, who were 6-year medical students and had successfully completed an internal medicine round. The students underwent training that included a theoretical session and practice for approximately 1 hour, in which the students performed at least 3 blood withdrawal procedures under the supervision of the lead researcher. For each study participant, in addition to the routine blood sample taken (the morning after the patient’s admission) by DVS, blood samples were drawn from PVC. If 20G PVC was used, blood was sampled again the next morning. A standard 23G needle attached to a vacutainer was used for sampling blood via DVS from the opposite limb as that with the PVC. Blood was drawn from PVC within 10 minutes of drawing from the DVS. The blood sampling method was identical for all participants: PVC was washed with 2 mL saline, with a 20-second wait period, followed by vein blocking for 15 seconds; a 5-mL syringe was attached to the PVC; a 5-mL blood “dead space” was drawn (and discarded in a medical waste), followed by insertion of a new 10-mL syringe and drawing 10 mL of blood, connection of an 18G needle, and injection of blood into 3 test tubes labeled as follows: 1-coagulation, 2-chemistry, and 3-CBC. Subsequently, PVC was washed with 2 mL saline and closed. If the patient received active infusion during the test, the infusion was stopped for 2 minutes, blood was drawn, and the infusion was resumed. In total, 15 mL of blood was drawn from the PVC and divided into 3 test tubes, as indicated above, in addition to the 3 tubes with DVS samples. After blood sampling, each collector filled a questionnaire, in which the following data were recorded: date and hour of sampling, PVC location on patient body, number of days since catheter insertion (1 or 2 days), PVC diameter (20G/22G), success/failure in blood sampling, prior sampling from the patient in the study, time elapsed since the last infusion (≥1 hour), and infusion type. Failure was defined as the lack of success in drawing blood or when the drawing time exceeded 90 seconds. Active infusion was defined as the use of PVC for drug or fluid administration at the time of blood sampling or ≥ 1 hour prior. PVC age was defined as day 1 when the test was performed in the morning after the patient’s arrival to the department (or if a new PVC was inserted the day before) and day 2 to indicate sampling on the morning of the following day. A sticker was attached to each questionnaire, with the participants identifying the information. Both tube sets from each patient were sent to the laboratory under identical conditions. Tests were performed using the same machine. Blood chemistry test tubes were tested using an OLYMPUS AU5800 analyzer (Beckman Coulter, Nyon, Switzerland), CBC tests were performed using an ADVIA 2120i analyzer (Siemens, Germany), and coagulation parameters were tested using a ROTINA 420R centrifuge (Ettich, Germany). Patients with missing data were excluded from this study.

### 1.4. Variables

The dependent variables included routine blood test results in a hospital setting for both DVS and PVC sampling methods (CBC parameters: hemoglobin [g/dL], platelet count [10^3^/µL], white blood cell count [10^3^ cells/µL]), chemical testing parameters (Na [mEq/L], K [mEq/L], Ca [mEq/dL], urea [mg/dL], creatinine [mg/dL], total bilirubin [mg/dL], aspartate aminotransferase [AST] [units/L], alanine aminotransferase [ALT] levels [U/L]), and measures of coagulation function (prothrombin time [PT] [s] and international normalized ratio).

The other dependent variables included hemolysis and failure rates. Hemolysis observed in the blood chemistry test was divided into 4 levels of severity: level 1 hemolysis invalidated the AST index, and levels 2, 3, and 4 invalidated the AST, K, and total bilirubin indices. Hemolysis levels were defined based on the hemoglobin level (mg/dL) in the blood chemistry test (Supplemental Table 1, http://links.lww.com/MD/G868). The primary outcome was the reliability of the laboratory indicators used to compare the 2 sampling methods (PVC vs DVS).

Secondary outcomes included the reliability of laboratory indicators used to compare PVC age (day 1 or 2 since insertion), PVC diameter (20G/22G), and extent of PVC use (active infusion/nonactive).

**Figure 1.**
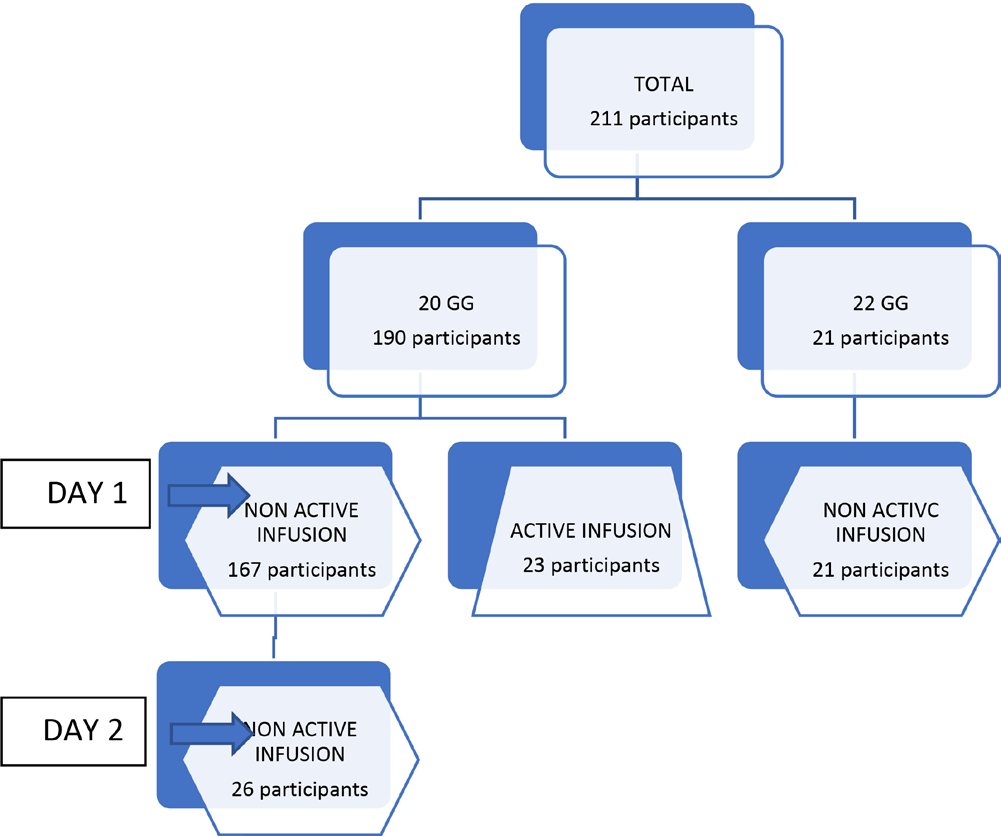
Study population divided into subgroups.

To examine the effects of each of the main independent variables, the study population was divided into several subgroups: day 1 group (the primary group of the study), day 2 group, active infusion group, and 22G group (Figure 1- Population division flow chart).

### 1.5. Statistical analysis

Dichotomous variables are presented as frequencies and percentages, and continuous variables are presented as means and standard deviations. Laboratory values for the PVC samples (labeled “research”) were compared with those of the DVS samples.

The Bland–Altman method was used to determine the degree of agreement between the tests for each laboratory value, and a 95% agreement interval was calculated. For each laboratory value, we used 3 methods to determine the clinically accepted interval (CAI). In the first method, CAI was set by the primary investigator together with the clinical staff of the Internal Medicine Department E, which is similar to the approach used by most previous studies.^[8,12,15]^ CAIs were determined so as not to exceed the combined intervals set by these central studies in the field and the Clinical Laboratory Improvement Amendments (CLIA) standards.^[8,10,12,15]^. Any calculated agreement interval found to be entirely within the limits of the CAI determined by both methods was defined as equivalent. A 95% acceptance interval (mean difference between the 2 methods ± standard deviation of 1.96) was calculated and compared with the CAI limits. The second approach involved the determination of CAIs, as described in other studies. CLIA standards were considered.^[10,15]^ CLIA represents the United States of America Federal Regulatory Standards that apply to all clinical laboratory tests performed in patients in the United States. These federal guidelines determined the maximum allowable analytical error for laboratories (Supplemental Table 2, http://links.lww.com/MD/G868).

The third method determines the degree of correlation between the tests and is involved in determining the intraclass correlation coefficient (ICC), which was calculated using SPSS version 25 (SAS Institute, Inc., Cary, NC, USA). The proportion of tests showing differences in results from all performed tests are listed under the heading “proportion” in the tables. The 95% confidence interval (CI) for the proportion of tests showing significant differences was calculated using the Clopper–Pearson exact method. P-values were calculated using Fisher exact test to determine if there were significant differences in hemolysis and failure rates between the 2 methods.

### 1.6. Ethical considerations

This study was approved by the institutional ethics committee. Each participant signed an informed consent form, and all patient information was kept confidential.

## 2. Results

The study population comprised 211 patients, of whom 26 were tested twice. There were no excluded participants due to missing data. The mean age was 60 years, and 57.3% of the patients were men. Most patients had cardiovascular disease (25.6%) or respiratory disease (16.6%). The most common PVC locations were the antecubital fossa (47.9%) and forearm (38.4%) (Table 1).

**Table 1:** Characteristics of the research population (n = 211).

Age (yr)	60 ± 18
Male (n, %)	121 (57.3%)
Anticoagulation/antiplatelet therapy:	
Anticoagulation	31 (14.6%)
Antiplatelets	58 (27.4%)
Anticoagulation + Antiplatelets	6 (2.8%)
Without treatment	108 (51.1%)
Unknown	8 (3.7%)
Diagnosis at admission:	
Cardiovascular	54 (25.6%)
Respiratory	35 (16.6%)
Genitourinary	14 (6.6%)
Gastrointestinal	13 (6.2%)
Hematologic	12 (5.7%)
Musculoskeletal	8 (3.8%)
Other	75 (35.5%)
Location of PVC:	
Antecubital fossa	101 (47.9%)
Forearm	81 (38.4%)
Back of the hand	28 (13.3%)

This study included a total of 237 blood tests. Of these, 167 were in the day 1 group, and the remaining 26 patients underwent a second blood test on day 2 (day 2 group). Twenty-one participants had 22G PVC, and 23 participants with 20G PVC had active infusion. Thirty tests (18%) of the day 1 group, 4 of the day 2 group (15.3%), one of the active infusion group (4.3%), and 2 of the 22G group (9.5%) were defined as failures. No significant differences were found among failure rates when each subgroup was compared with the primary group (day 1 group, which was a 20G, nonactive infusion group) (Table 2).

**Table 2:** Failure rates.

Group	Day 1 (n = 167)	Day 2 (n = 26)	*P* value
Failure rate (N,%)	30 (17.96%)	4 (15.38%)	1.000
Group	Nonactive infusion (n = 167)	Active infusion (n = 23)	*P* value
Failure rate	30 (17.96%)	1 (4.34%)	0.133
Group	20G (n = 167)	22G (n = 21)	*P* value
Failure rate	30 (17.96%)	2 (9.5%)	0.538

ALT = alanine aminotransferase, AST = aspartate aminotransferase, CAI = clinically accepted interval, 95% AI, 95% agreement interval according to the Bland–Altman analysis, CBC = complete blood count, ICC = intraclass correlation coefficient, INR = international normalized ratio, Number of tests = number of tests with valid findings for direct venipuncture stab (DVS) and peripheral venous catheter (PVC), PT = prothrombin time, Tests showing difference = number of tests showing differences between DVP and PVC greater than the interval defined (clinically accepted interval [CAI] as defined by physicians’ consensus or agreement interval that was calculated according to Bland–Altman analysis), WBC = white blood cell.

Overall, 200 blood samples were collected. In the day 1 group, 2 samples (1.5%) showed level 1 hemolysis, and none showed hemolysis at the other levels with DVS sampling. In the same group, hemolysis occurred in 15 samples (8 level 1 [5.8%], 4 of level 2 [2.9%], one of level 3 [0.7%], and 2 of level 4 [1.5%]) with PVC sampling. No significant differences were found between the 2 methods at any level. The hemolysis rate for all levels was 1.47% with DVS sampling and 10.9% with PVC sampling, which were significantly different (*P* = .002) (Table 3).

**Table 3:** Correlation, concordance, and equivalence between the 2 blood sampling methods on day 1 (DVS vs PVC).

				Clinically accepted interval				Agreement interval				
Determination	Variable	Number of tests	ICC	CAI	Tests showing differences	Proportion	95% CI*	95% AI	Tests showing differences	Proportion	95% CI*	Methods (PVC vs DVS) considered interchangeable
CBC	Hemoglobin level (g/dL)	134	0.988	± 1	2	0.0149	0.002–0.05	−0.78–1.01	2	0.149	0.002–0.05	Yes
	WBC count (103 cells/µL) (A-4)	134	0.995	± 1.5	2	0.0149	0.002–0.05	−1–1.1	7	0.0522	0.0213–0.1047	Yes
	Platelet count (103/µL)	132	0.977	± 50	5	0.0379	0.0124–0.0862	−47.1–41.6	6	0.0455	0.0169–0.0963	Yes
Basic chemical analysis	Sodium level (mEq/L)	135	0.954	± 4	0	0	0.00–0.027	−2.6–3	4	0.0296	0.0081–0.0741	Yes
	Potassium level (mEq/L) (A-5)	128	0.941	± 0.45	5	0.0391	0.0128–0.088	−0.35–0.45	6	0.0469	0.0174–0.0992	Yes
	Calcium level (mEq/dL)	129	0.970	± 1	0	0	0.00–0.0282	−0.37–0.46	7	0.0543	0.0543	Yes
	Urea level (mg/dL)	135	0.999	± 5	0	0	0.00–0.0027	−1.8–2.2	9	0.0667	0.0309–0.1228	Yes
	Creatinine (mg/dL)	135	0.998	± 0.2	0	0	0.0002–0.0027	−0.09–0.11	8	0.0593	0.0259–0.1134	Yes
	Total bilirubin (mg/dL)	126	0.999	± 0.2	1	0.0079	0.0002–0.0434	−0.14–0.16	1	0.0079	0.0002–0.1434	Yes
	ALT (units/L)	132	0.999	± 10	0	0	0.00–0.0276	−3.3–3.2	7	0.0530	0.0216–0.1062	Yes
	AST (units/L)	122	0.998	± 10	0	0	0.00–0.0298	−4.2–3.8	6	0.0492	0.0183–0.1040	Yes
Coagulation indices:	PT-INR	135	0.997	± 0.11	0	0	0.00–0.027	−0.04–0.06	8	0.0593	0.0259–0.1134	Yes
	PT (s)	135	0.997	± 1.5	0	0	0.00–0.027	−0.45–0.66	7	0.0519	0.0211–0.1039	Yes

ALT = alanine aminotransferase, AST =aspartate aminotransferase, CAI = clinically accepted interval, CBC = complete blood count, ICC =intraclass correlation coefficient, 95% AI, 95% agreement interval according to the Bland–Altman analysis, INR = international normalized ratio, Number of tests = Number of tests with valid findings for direct venipuncture stab (DVS) and peripheral venous catheter (PVC), Tests showing difference = Number of tests showing differences between DVP and PVC greater than the interval defined (clinically accepted interval [CAI] as defined by physicians’ consensus or agreement interval that was calculated according to Bland–Altman analysis), PT = prothrombin time, WBC = white blood cell.

*95% confidence interval for the proportion of pairs showing a difference greater than the interval (n) was obtained using the Clopper–Pearson exact method.

On day 2, the total hemolysis rate was 9.4% with DVS sampling and 4.7% with PVC sampling. In the 22G group, 1 sample (5.3%) showed hemolysis with DVS sampling, whereas none showed hemolysis with PVC sampling (0%). In the active infusion group, 1 sample showed hemolysis with PVC sampling (4.5%), whereas 2 samples showed hemolysis with DVS sampling (9.5%). In the active infusion, day 2, and 22G groups, no significant differences were noted in each level or in the total hemolysis rate between the 2 methods (Table 4).

**Table 4:** Correlation, concordance, and equivalence between the 2 blood sampling methods on day 2 (DVS vs PVC).

				Clinically accepted interval					Agreement interval			
Determination	Variable	Number of tests	ICC	CAI	Tests showing differences	Propotion	95% CI*	95% AI	Tests showing differences	Propotion	95% CI*	Methods (PVC vs DVS) considered interchange able
CBC	Hemogln level (g/dL)(A-6)	20	0.994	±1	0	0	0.00–0.184	−0.73–0.68	1	0.05	0.0013–0.248	Yes
	WBC count (103 cells/μL)	21	0.995	±1.5	0	0	0.00–0.1611	–0.38–0.8	1	0.0522	0.0213–0.1047	Yes
	Platelet count (103/μL)	21	0.989	±50	0	0	0.00–0.1611	–34.5–43.7	1	0.0522	0.0213–0.1047	Yes
Basic chemical analysis indices:	Sodium level (mEq/L)	21	0.960	±4	0	0	0.00–0.1611	–2.6–2.6	1	0.0522	0.0213–0.1047	Yes
	Potassium level (mEq/L)	20	0.913	±0.45	1	0.05	0.0013–0.248	–0.19–0.44	1	0.05	0.0013–0.248	Yes
	Calcium level (mEq/dL)	21	0.972	±1	0	0	0.00–0.1611	–0.16–0.39	2	0.0952	0.0117–0.3038	Yes
	Urea level (mg/dL)	21	0.999	±5	0	0	0.00–0.1611	–2.1–2.7	1	0.0522	0.0213–0.1047	Yes
	Creatinine (mg/dL)	21	0.997	±0.2	0	0	0.00–0.1611	–0.08–0.05	1	0.0522	0.0213–0.1047	Yes
	Total bilirubin (mg/dL)	20	0.999	±0.2	0	0	0.00–0.184	–0.04–0.16	3	0.15	0.0321–0.3789	Yes
	ALT (units/L)	21	0.999	±10	0	0	0.00–0.1611	–1.1–2.5	1	0.0522	0.0213–0.1047	Yes
	AST (units/L)	20	0.999	±10	0	0	0.00–0.184	–2.7–4.5	0	0	0	Yes
Coagulation PT-INR 21 indices:	PT-INR	21	0.996	±0.11	0	0	0.00–0.1611	–0.04–0.04	1	0.0522	0.0213–0.1047	Yes
	PT (seconds)	21	0.997	±1.5	0	0	0.00–0.1611	–0.41–0.46	2	0.0922	0.0117–0.3038	Yes

ALT = alanine aminotransferase, AST =aspartate aminotransferase, 95% AI = 95% agreement interval according to the Bland–Altman method, CAI = clinically accepted interval, CBC = complete blood count, PT = prothrombin time, ICC =intraclass correlation coefficient, INR = international normalized ratio, Number of tests = number of tests with valid findings for direct venipuncture stab (DVS) and peripheral venous catheter (PVC), Tests showing difference = number of tests showing differences between DVPand PVC greater than the interval defined (clinically accepted interval [CAI] as defined by physicians’ consensus or agreement interval that was calculated according to the Bland–Altman method), WBC = white blood cell.

*95% confidence interval for the proportion of pairs showing a difference greater than the interval (n) was obtained using the Clopper–Pearson exact method.

ICC indicated a significant correlation among all indices in all groups (Tables 3–4 and supplemental 3-4, http://links.lww.com/MD/G868).

Both methods showed equal reliability for all variables examined using the Bland–Altman method for all groups. The only measures that exceeded CAI were hemoglobin in 2 of the groups: in the day 1 group with a deviation of 0.1 g/dL from CAI (CAI ± 1, 95% CI, AI: −0.78 to 1.01) and in the active infusion group with a deviation of 0.4 g/dL from CAI (CAI ± 1, 95% CI, AI: −0.86 to 1.4) (Tables 3–4 and supplemental tables 3–4, http://links.lww.com/MD/G868).

## 3. Discussion

This study examined the reliability of common laboratory blood test results of blood samples obtained using 2 PVC and DVS sampling methods. The study showed a strong agreement between the 2 methods in numerous patients using several indicators taken from routine daily laboratory tests at the hospital laboratory. These trial results should alleviate the concerns related to PVC blood sampling, including high failure and hemolysis rates. Moreover, this study showed that the reliability of PVC blood sampling is not negatively affected by catheter age, diameter, or use for the delivery of fluids and medications.

Surprisingly, this comparison between the 2 sampling methods has rarely been investigated. Most published studies have included small populations,^[1–3]^ only a few indicators, and tested no more than 1 independent variable. Although most indicators showed high reliability in terms of laboratory results for both DVS and PVC samplings, these studies did not report on failure or hemolysis rates. For example, 1 study determined that blood test results with PVC sampling (after a 5-mL dead space was discarded into medical waste) were more reliable than those with DVS sampling; the study included only 60 participants and referred to only 4 indicators (sodium, potassium, urea, and creatinine).^[9]^ Only 1 study examined the effects of time elapsed following PVC insertion on blood test results and showed that PVC could be used after 1 and 2 days of insertion, but it only examined coagulation functions (PT + PTT)^[14]^.

Notably, our study included a relatively large number of participants (n = 211). The importance of the sample size was elucidated in a systematic review by Lesser et al ^[16]^. The studies by Corbo et al, [10]Zlotowski et al, ^[12]^ and Berger–Achituv et al ^[15]^ included 31 to 81 participants. The studies that included a large number of participants, such as those by Ortells–Abuye et al ^[8]^ and Hambleton et al, ^[11]^ which included 272 and 258 participants, respectively, did not include important variables that affect the reliability of blood test results on samples obtained using a PVC, such as active infusion and age of the PVC. Apart from the study by Herr et al, ^[7]^ which included the PVC failure rate, all other studies excluded failed attempts at PVC sampling. We believe that the failure rate, which was relatively low in our study (18% in the day 1 group, 15.3% on day 2, 4.3% at active infusion, and 9.5% in the 22G group), is a critical consideration in making the decision to withdraw blood using a PVC.

Our study demonstrated the reliability of PVC sampling, including when PVC is used for infusion or has a different needle gauge and on the first and second days after PVC insertion. The only measure that exceeded the clinically acceptable interval was hemoglobin in the day 1 and active infusion groups, with deviations of + 0.1 and + 0.4, respectively, from CAI, which may be considered as minimal and not significant clinically acceptable deviations. Although the hemolysis rate in PVC blood samples drawn on day 1 was significantly higher than that in the DVS sample, it was still among the lowest compared with those previously reported.^[6,5]^ In addition, the main degree of hemolysis in the PVC blood sample drawn on day 1 was level 1, which invalidated only AST test results. We believe that this relatively lower rate was achieved because of our withdrawal study protocol and the use of a syringe for controlled blood aspiration.

There were no significant differences in failure rates between the 2 groups. The failure rate in the day 1 group (the primary group of the study) was 17%. We believe that this is an acceptable rate that makes PVC sampling over DVS sampling, which may require several attempts to find a vein, thus unnecessarily causing pain. Most patients in whom PVC blood sampling was unsuccessful remarked that the catheter had not been washed with saline in the emergency room before insertion, and a clot was clearly visible in the catheter opening. Failures on day 2 were mainly observed in patients in whom the catheter was not washed after infusion in the ward. We believe that washing the PVC properly can minimize the failure rate with ease.

We did not examine the effects of the anatomic location of PVC on failure rates, as done in previous studies, which showed that the antecubital vein was superior to other sites. In our study, almost half of the PVC samples were obtained from smaller veins, and the results remained consistent.

The main goal of this study was to determine whether PVC blood sampling is reliable in patients hospitalized for several days in the Internal Medicine Department without the need for repeated venipunctures, especially in patients with limited venous access, thus saving the patient from pain and the valuable work time of the healthcare staff. PVC sampling may increase patient responsiveness by reducing the number of invasive procedures. It also decreases the risk of contamination by healthcare workers and collateral spreading of contagious diseases by reducing unnecessary exposure, especially at times such as the present when maintaining contact isolation between patient and staff is necessary due to COVID-19.

There were major limitations to our study. This was a single-center trial performed in a single department. The diameters of the 2 catheters were compared. The sample power was calculated only for the primary outcome; therefore, the statistical strength was decreased for secondary outcomes, although it was compensated for by the larger sample size than had been planned. Nevertheless, this study included a relatively large population, and despite the limitations, the results were statistically robust.

Another major drawback of our study was that we did not examine the troponin blood and blood gas tests, which, according to Lesser et al,^[16]^ are not reliable tests when the sample is obtained using a PVC.

Our findings have led us to change the protocols used in our department, and PVC blood sampling is now frequently used for routine laboratory blood tests. A larger multicenter trial should be performed to strengthen our results and encourage global policy changes.

## 4. Conclusions

Both blood sampling methods (via PVC or DVS) can be used interchangeably for the majority of routine laboratory tests on days 1 and 2 after PVC insertion using 20G- or 22G PVC; sampling can also be done in the case of infused PVC. Washing the PVC properly can easily minimize the failure rate in blood samples.

## Author contributions

Idan Ben Shabat-: initiator of study part of the team performing the trial collecting data writing the article. Michal Hoffman: part of the team performing the trial collecting data writing the article.

Shai Ben Abraham: part of the team performing the trial collecting data writing the article.

Iftach Sagy: Statistical analysis reviewer of the article. Gal Tsaban: Statistical analysis Writing the article. Merav Cohen- Lahav: Quality control of laboratory examinations performing laboratory exams. Michal Peled: performing laboratory exams. Carmi Bartal: Primary Investigator writing and reviewing the article.

## Supplementary Material


