# Supplementary material for: Reliability of blood tests taken from the peripheral intravenous catheter
Source: Medicine (Baltimore). 2022 Jul 15;101(28):e29268. doi: 10.1097/MD.0000000000029268 (PMC11132390; doi:10.1097/MD.0000000000029268)
Supplement: Supplementary file 1 [file medi-101-e29268-s001.pdf]

Supplemental Digital Content

Supplentary Table 1- Hemolysis estimation (Approximate concentration of chromatic substances)

**Flag**	**HEM (mg/dL hemoglobin)**
+	50-99
++	100-199
+++	200-299
++++	300-500

Table A. 2. The criteria for acceptable performance by CLIA standards as of November 2018.

**Analyte or test**	**Criteria for acceptable performance**
Hemoglobin	Target ±7%.
Leukocyte count	Target ±15%.
Platelet count	Target ±25%.
Prothrombin time	Target ±15%.
Sodium	Target value ±4 mmol/L.
Potassium	Target value ±0.5 mmol/L.
Calcium, total	Target value ±1.0 mg/dL.
Urea nitrogen	Target value ±2 mg/dL or ±9% (greater).
Creatinine	Target value ±0.3 mg/dL or ±15% (greater).
Bilirubin, total	Target value ±0.4 mg/dL or ±20% (greater).
Alanine aminotransferase (ALT/SGPT)	Target value ±20%.
Aspartate aminotransferase (AST/SGOT)	Target value ±20%.

Table A. 3. Hemolysis rates

Hemolysis level	Day 1			Day 2			Active infusion			22G		
	Venipuncture (N=136)	PVC (N=137)	P value	Venipuncture (N=21)	PVC (N=21)	P-value	Venipuncture (N=21)	PVC (N=22)	P value	Venipuncture (N=19)	PVC (N=19)	P-value
1	2 (1.47%)	8 (5.8%)	0.103	1 (4.7%)	1 (4.7%)	1	1 (4.7%)	1 (4.5%)	1	0	0	-
2	0	4 (2.9%)	0.122	1 (4.7%)	0	1	0	0	-	1 (5.3%)	0	1
3	0	1 (0.7%)	1	0	0	-	0	0	-	0	0	-
4	0	2 (1.5%)	0.498	0	0	-	1 (4.7%)	0	0.488	0	0	-
Total percentage	2 (1.47%)	15 (10.9%)	0.002	2 (9.4%)	1 (4.7%)	0.52	2 (9.5%)	1 (4.5%)	0.607	1 (5.3%)	0	1

Figure A. 1

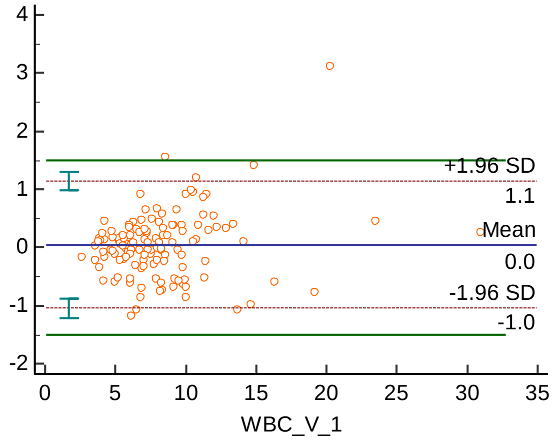

Bland altman plot for WBC at day 1 group. A-4

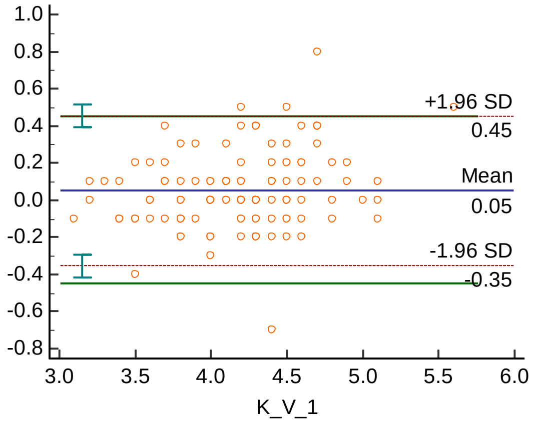

Bland altman plot for K at day 1 group.A-5

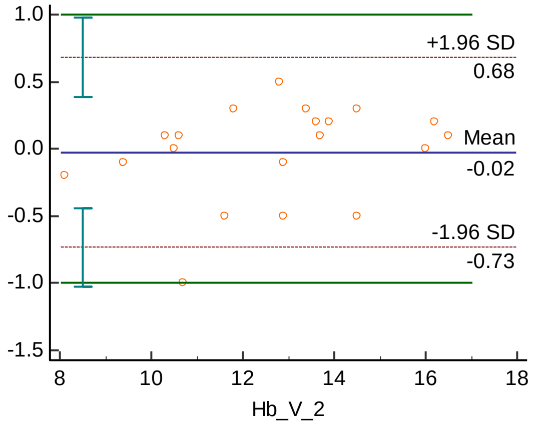

Bland altman plot for HB at day 2 group.A-6

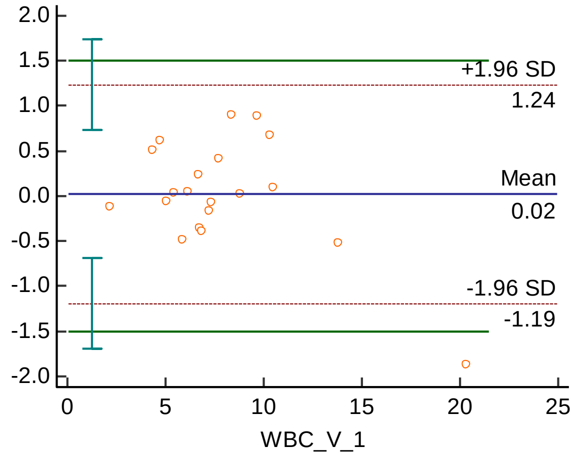

Bland altman plot for WBC at active infusion group.A-7

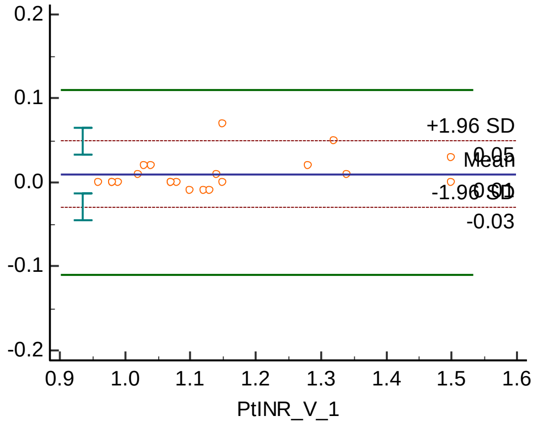

Bland altman plot for PT-INR at active infusion group. A-8

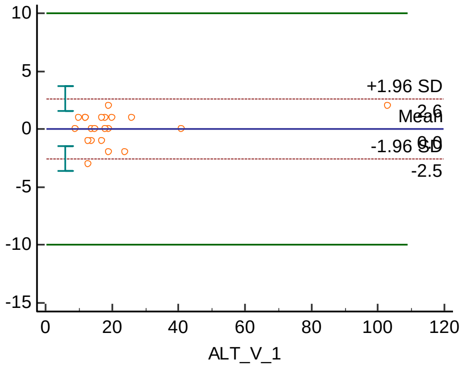

Bland altman plot for ALT at active infusion 9group.A-

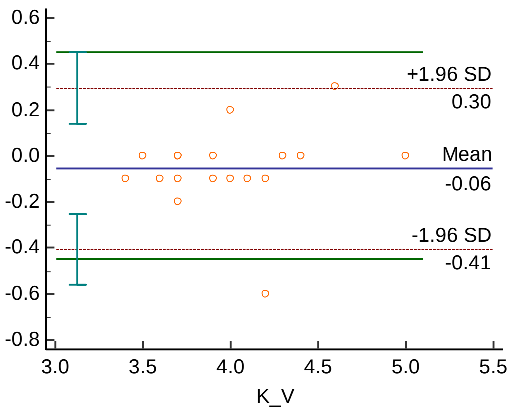

Bland altman plot for K at 22G infusion group. A-10

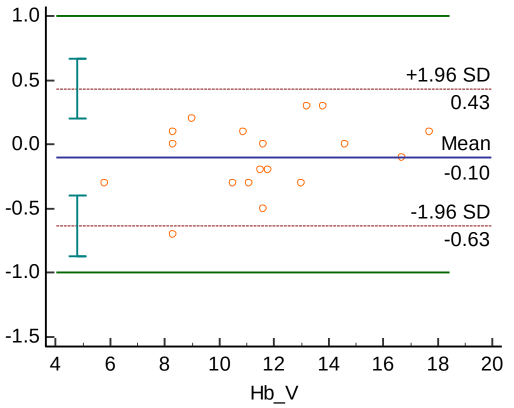

Bland altman plot for HB at 22G infusion group. A-11

Supplemental Digital Content

Supplemental Table 2- The Clinical Laboratory Improvement Amendments (CLIA) standards 2018**.**

Supplemental Table 2. The criteria for acceptable performance by CLIA standards current as of 2018.

Analyte or test	Criteria for acceptable performance
Hemoglobin (gr/dL)	Target ±7%.
White blood cells (10^3/ul)	Target ±15%.
Platelet count (10^3/ul)	Target ±25%.
Prothrombin time (sec)	Target ±15%.
Partial Thrombopiastin Time (sec)	Target ±15%.
Sodium (mEq/L)	Target value ±4.
Potassium (mEq/L)	Target value ±0.5.
Calcium, total (mg/dL)	Target value ±1.0.
Urea nitrogen (mg/dL)	Target value ±2 mg/dL or ±9% (greater).
Creatinine (mg/dL)	Target value ±0.3 mg/dL or ±15% (greater).
Bilirubin, total (mg/dL)	Target value ±0.4 mg/dL or ±20% (greater).
Alanine aminotransferase (ALT/SGPT) (U/L)	Target value ±20%.
Aspartate aminotransferase (AST/SGOT)	Target value ±20%.

CLIA represents the United States of America Federal Regulatory Standards that apply to all clinical laboratory tests performed in patients in the United States. These federal guidelines determine the maximum allowable analytical error for laboratories.

Supplemental Digital Content

Supplemental Table 3: Correlation, concordance, and equivalence between the two blood sampling methods in the 22G group (DVS vs PVC).

Determination	Variable	Number of tests	ICC	Clinically accepted interval				Agreement interval				
				CAI	Tests showing differences	Proportion	95% CI	95% AI	Tests showing differences	Proportion	95% CI	Methods (PVC vs DVS) considered interchangeable
**CBC**	Hemoglobin (g/dL)(A-11)	18	0.998	± 1	0	0	0–0.185	–0.633–0.433	1	0.005	0.001–0.272	Yes
	WBC count (10^3 cells/µL)	18	0.998	± 1.5	0	0	0–0.185	–1.381–1.07	0	0	0–0.185	Yes
	Platelet count (10^3 /µL)	18	0.992	± 50	0	0	0–0.185	–19.77–30.55	1	0.005	0.001–0.272	Yes
**Basic chemical analysis indices**:	Sodium level (mEq/L)	19	0.979	± 4	0	0	0–0.176	–2.012–2.43	1	0.0526	0.0013–0.26	Yes
	Potassium level (mEq/L)(A-10)	18	0.949	± 0.45	1	0.005	0.001–0.272	–0.205–0.4	2	0.105	0.013–0.33	Yes
	Calcium level (mEq/dL)	19	0.975	± 1	0	0	0–0.176	–0.43–0.52	2	0.105	0.013–0.33	Yes
	Urea level (mg/dL)	19	0.999	± 5	1	0.0526	0.0013–0.26	–4.4–4	1	0.0526	0.0013–0.26	Yes

	Creatinine (mg/dL)	19	0.997	± 0.2	0	0	0–0.176	–0.11–0.1	1	0.0526	0.0013–0.26	Yes
	Total bilirubin (mg/dL)	18	1	± 0.2	0	0	0–0.185	–0.047–0.06	1	0.005	0.001–0.272	Yes
	ALT (units/L)	19	1	± 10	1	0.0526	0.0013–0.26	–6–5.1	1	0.0526	0.0013–0.26	Yes
	AST (units/L)	18	1	± 10	0	0	0–0.185	–4.7–3.9	1	0.005	0.001–0.272	Yes
**Coagulation indices**:	PT-INR	19	0.995	± 0.11	0	0	0–0.176	–0.04–0.05	1	0.0526	0.0013–0.26	Yes
	PT (seconds)	19	0.996	**± 1.5**	0	0	0–0.176	–0.51–0.6	2	0.105	0.013–0.33	Yes

Number of tests, Number of tests with valid findings for direct venipuncture stab (DVS) and peripheral venous catheter (PVC); Tests showing difference, Number of tests showing differences between DVS and PVC greater than the interval defined (clinically accepted interval [CAI] as defined by physicians' consensus or agreement interval that was calculated according to the Bland–Altman method); WBC, white blood cell; ALT, alanine aminotransferase; AST, aspartate aminotransferase; CBC, complete blood count; PT, prothrombin time; INR, international normalized ratio; ICC, intraclass correlation coefficient; CAI, clinically accepted interval; 95% AI; interval of agreement of 95% according to the Bland–Altman method

*95% confidence interval for the proportion of pairs showing a difference greater than the interval (n) was obtained using the Clopper–Pearson exact method.

Supplemental Digital Content

Supplemental Table 4-: Correlation, concordance, and equivalence between the two blood sampling methods in the active infusion group (DVS vs PVC).

Determination	Variable	Number of tests	ICC	Clinically accepted interval				Agreement interval				Methods (PVC vs DVS) considered interchangeable
				CAI	Tests showing differences	Proportion	95% CI	95% AI	Tests showing differences	Proportion	95% CI	
**CBC**	Hemoglobin level (g/dL)	20	0.983	± 1	2	0.1	0.0123–0.317	–0.86–1.4	1	0.05	0.0013–0.248	No
	WBC count (10^3 cells/μL)(A-7)	20	0.994	± 1.5	1	0.05	0.0013–0.248	–1.19–1.24	1	0.05	0.0013–0.248	Yes
	Platelet count (10^3/μL)	20	0.992	± 50	0	0	0.00–0.184	–27.8–25.5	2	0.1	0.0123–0.317	Yes
**Basic chemical analysis indies:**	Sodium level (mEq/L)	20	0.965	± 4	0	0	0.00–0.184	–2.6–2.5	0	0	0.00–0.184	Yes
	Potassium level (mEq/L)	19	0.939	± 0.45	0	0	0–0.1765	–0.45–0.45	0	0	0–0.1765	Yes
	Calcium level (mEq/dL)	21	0.975	± 1	0	0	0.00–0.1611	–0.45–0.5	2	0.0952	0.0117–0.3038	Yes
	Urea level (mg/dL)	21	0.999	± 5	1	0.0522	0.0213–0.1047	–2.5–3.4	1	0.0522	0.0213–0.1047	Yes

	Creatinine (mg/dL)	21	0.997	± 0.2	2	0.0952	0.0117–0.3038	–0.18–0.18	2	0.0952	0.0117–0.3038	Yes
	Total bilirubin (mg/dL)	20	0.998	± 0.2	0	0	0.00–0.184	–0.11–0.15	2	0.1	0.0123–0.317	Yes
	ALT (units/L) (A-9)	21	0.999	± 10	0	0	0.00–0.1611	–2.5–2.6	1	0.0522	0.0213–0.1047	Yes
	AST (units/L)	20	0.999	± 10	0	0	0.00–0.184	–5.9–6.9	3	0.15	0.0321–0.379	Yes
**Coagulation indices**:	PT-INR (A-8)	21	0.995	± 0.11	0	0	0.00–0.1611	–0.03–0.05	2	0.0952	0.0117–0.3038	Yes
	PT (sec)	21	0.994	**± 1.5**	0	0	0.00–0.1611	–0.48–0.67	2	0.0952	0.0117–0.3038	Yes

Number of tests, Number of tests with valid findings for direct venipuncture stab (DVS) and peripheral venous catheter (PVC); Tests showing difference, Number of tests showing differences between DVS and PVC greater than the interval defined (clinically accepted interval [CAI] as defined by physicians' consensus or agreement interval that was calculated according to the Bland–Altman method); ICC, intraclass correlation coefficient; CAI, clinically accepted interval; 95% AI; interval of agreement of 95% according to the Bland-Altman method; WBC, white blood cell; ALT, alanine aminotransferase; AST, aspartate aminotransferase; CBC, complete blood count; PT, prothrombin time; INR, international normalized ratio.

*95% Confidence interval for the proportion of pairs showing difference greater than the interval (n) was obtained using the Clopper–Pearson exact method.
